# Off-label ozempic and pancreatitis: legitimate concern or overblown fear?

**DOI:** 10.1097/MS9.0000000000003951

**Published:** 2025-09-22

**Authors:** Hafsa Ajmal, Hafiz Muhammad Moaaz Sajid, Ussama Shafqat, Hafiz Sohail Ashraf, Muddassir Khalid, Fred Segawa

**Affiliations:** a Department of Medicine, Mayo Hospital, Lahore, Pakistan; b Department of Medicine, Punjab Medical College, Faisalabad Medical University, Faisalabad, Pakistan; c Carle Foundation Hospital, Urbana, Illinois, USA; d Department of Medicine, Nishtar Medical University, Multan, Pakistan; e Department of Medicine, Makerere University College of Health Sciences, Kampala, Central Region, Uganda

Semaglutide, a potent glucagon-like peptide-1 (GLP-1) receptor agonist, has attracted considerable interest as a pharmacological option for weight loss. Although semaglutide was initially developed and approved for managing type 2 diabetes, its use at higher doses for obesity without diabetes was established through large randomized controlled trials (add reference). The U.S. Food and Drug Administration approved semaglutide 2.4 mg weekly (Wegovy®) for long-term weight management in 2021. Conversely, lower doses of semaglutide (Ozempic®) remain indicated for glycemic control but are often prescribed off-label for weight loss. Both indications list pancreatitis as a rare but potentially serious adverse event in the prescribing information, warranting discontinuation if suspected^[1]^. The risk of acute pancreatitis in non-diabetic individuals using semaglutide for weight loss has become an increasing concern in both clinical and regulatory spheres. Data from randomized controlled trials involving obese populations suggest that pancreatitis events are infrequent and comparable to those in the placebo group. For instance, in the pivotal STEP programme, adjudicated pancreatitis occurred at a rate of approximately 0.2 cases per 100 patient-years, and no cases were observed in the 2-year extension trial across treatment and placebo groups^[1,2]^. These results support the idea that pancreatitis is rare in large, well-conducted clinical trials, although such studies may lack the power to detect very infrequent safety signals. More recently, pharmacoepidemiological studies have attempted to quantify this risk in real-world weight loss settings.

A 2023 JAMA study limited to non-diabetic adults prescribed GLP-1 receptor agonists for weight loss found that semaglutide users had a higher relative risk of pancreatitis compared to those receiving naltrexone-bupropion, though absolute case numbers remained low. While this analysis grouped semaglutide with liraglutide, it is noteworthy that semaglutide accounts for the majority of current prescriptions and off-label weight-loss use, making these findings relevant to its safety profile in this context. Significantly, observational studies may be confounded by baseline risk factors, but the signal appears consistent enough to warrant clinical vigilance^[3]^. Case reports offer more detailed evidence of pancreatitis associated with semaglutide used solely for weight loss in non-diabetic individuals. Hughes *et al* described a 36-year-old woman who developed acute pancreatitis shortly after starting semaglutide for cosmetic weight loss, with other causes excluded and resolution following discontinuation^[4]^.

Another 2024 case report in the American Journal of Gastroenterology detailed a patient who developed pancreatitis after beginning semaglutide for weight loss, again without alternative causes identified^[5]^. These reports emphasize that although rare, pancreatitis can occur in otherwise low-risk, non-diabetic individuals using semaglutide for weight management. Regulatory authorities have also recognized this emerging issue. The UK Medicines and Healthcare products Regulatory Agency recently announced a focused safety review into serious harms, including pancreatitis, linked to GLP-1 agonists amid their increasing use in non-diabetic populations for weight reduction^[6]^. This underscores the global recognition of the importance of post-marketing surveillance as prescribing practices evolve toward weight-loss indications. Overall, current evidence indicates that acute pancreatitis in non-diabetic patients using semaglutide for weight loss is uncommon but biologically possible^[2]^. Randomized controlled trials report a very low incidence. At the same time, observational data suggest a potential relative increase in risk compared to other agents, and case reports confirm its occurrence in real-world settings^[4,5]^. For clinicians, this risk necessitates careful patient selection, counseling about early warning signs such as persistent severe epigastric pain, and prompt discontinuation if pancreatitis is suspected. Until more definitive data become available, vigilance remains essential, especially given the increasing off-label use of semaglutide for cosmetic weight loss^[1,6]^.

TITAN Guidelines: This manuscript is compliant with TITAN Guidelines, 2025, declaring no use of AI^[7]^.

## Data Availability

Not Applicable.
